# Supplementary figures and images for: A simple regulatory architecture allows learning the statistical structure of a changing environment
Source: eLife. 2021 Sep 7;10:e67455. doi: 10.7554/eLife.67455 (PMC8423446; doi:10.7554/eLife.67455)

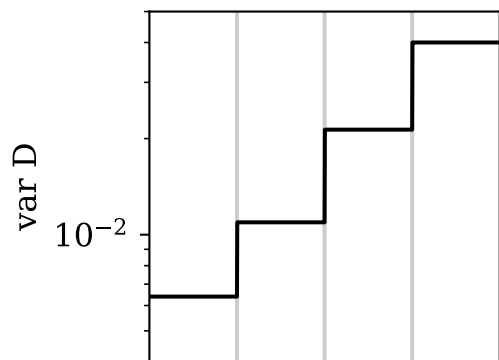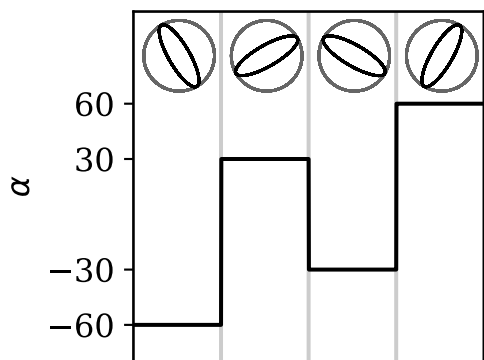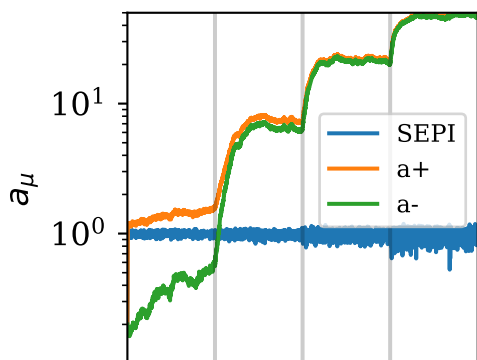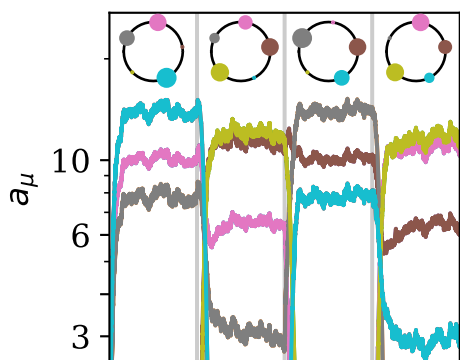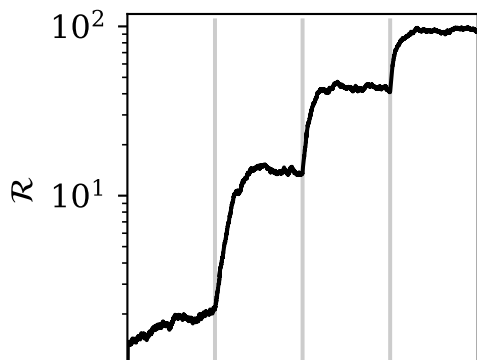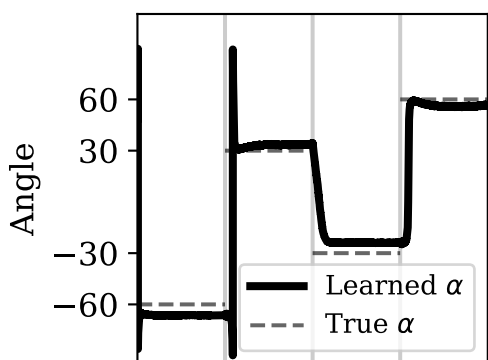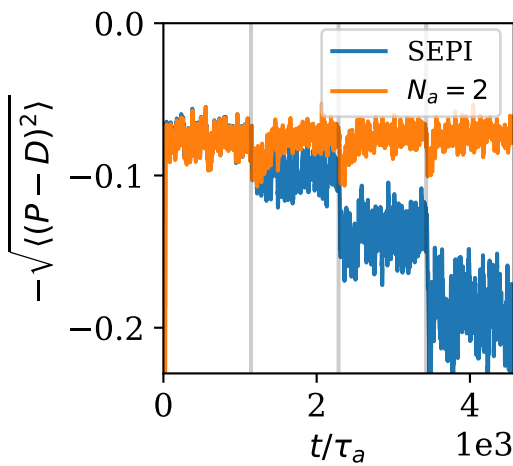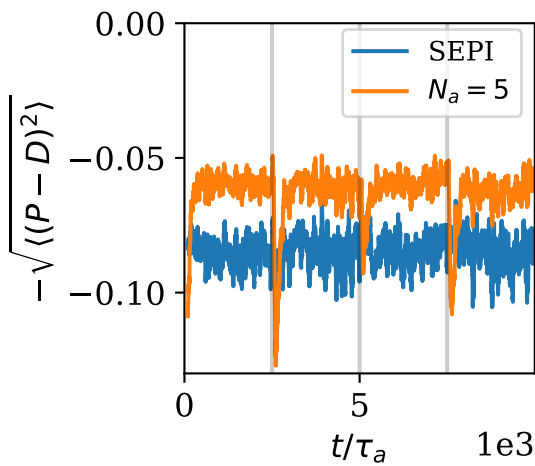

Supplement: Source code 1. [file elife-67455-code1.zip › data_and_scripts/Figure 3/fig3.pdf]

**A**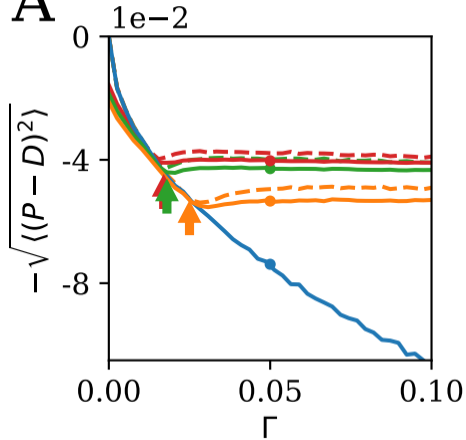**B**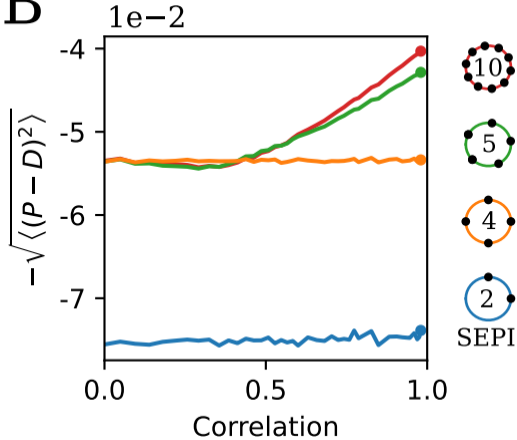

Supplement: Source code 1. [file elife-67455-code1.zip › data_and_scripts/Figure 4/fig4.pdf]

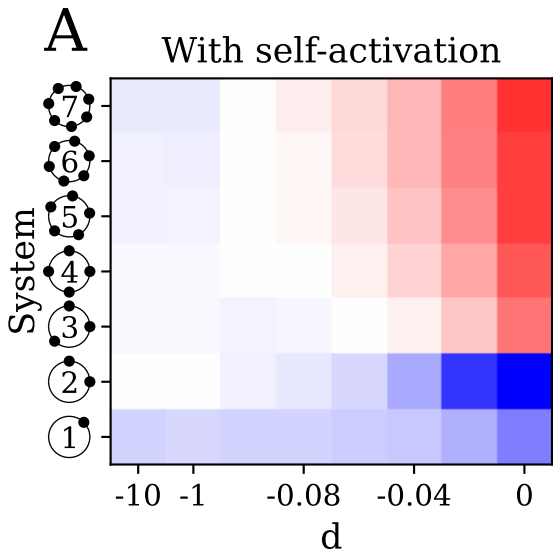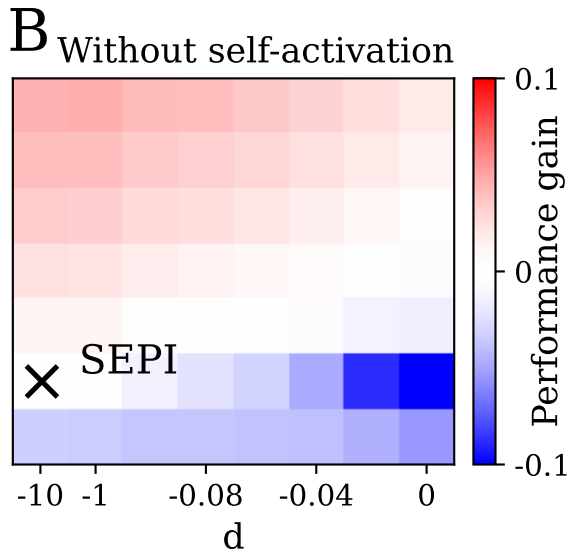

Supplement: Source code 1. [file elife-67455-code1.zip › data_and_scripts/Figure 5/fig5.pdf]

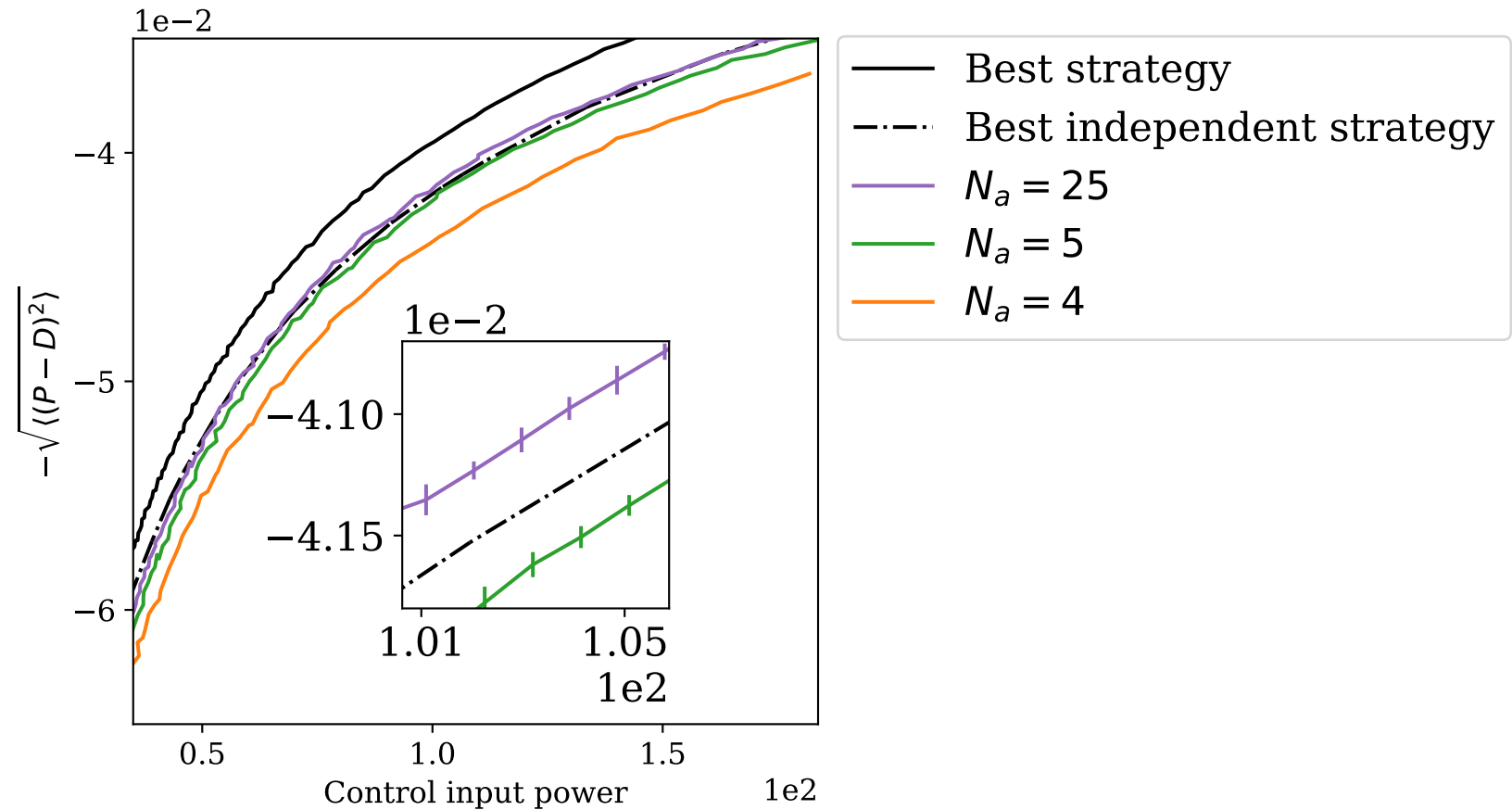

Supplement: Source code 1. [file elife-67455-code1.zip › data_and_scripts/Supplement/S1/FigS1.pdf]

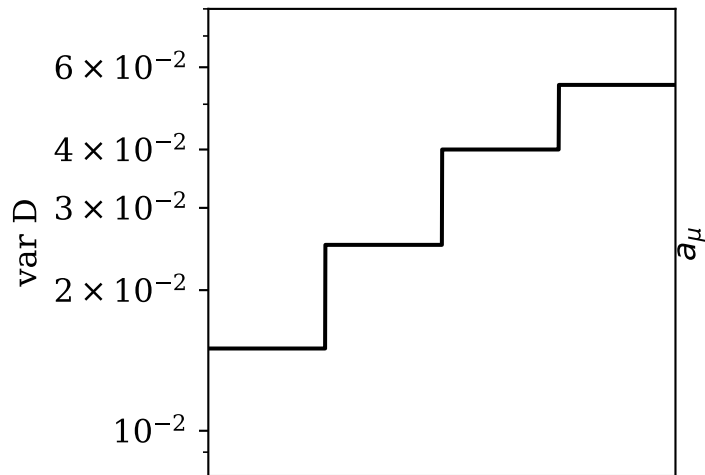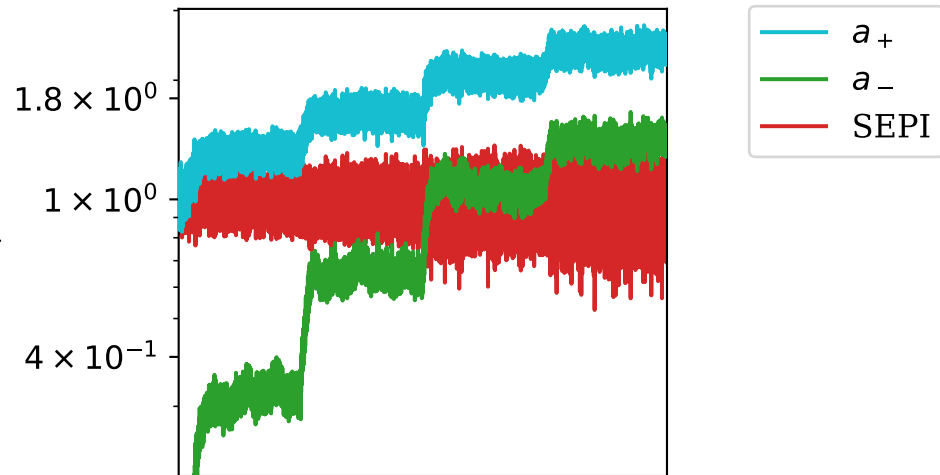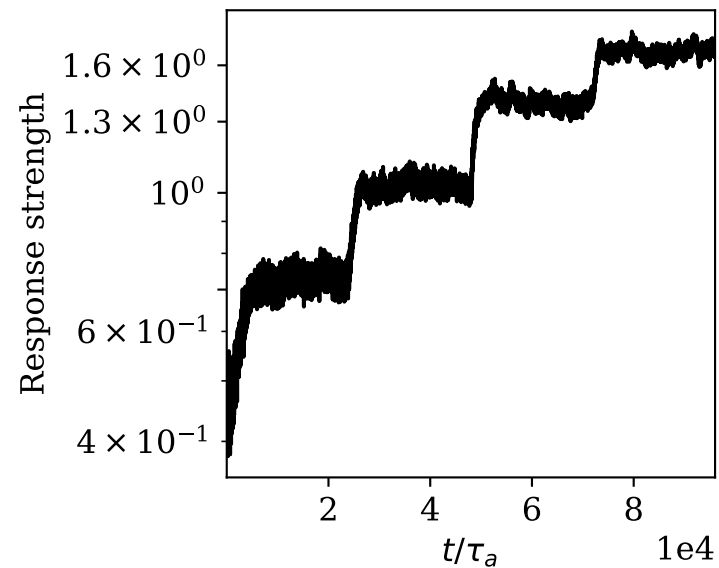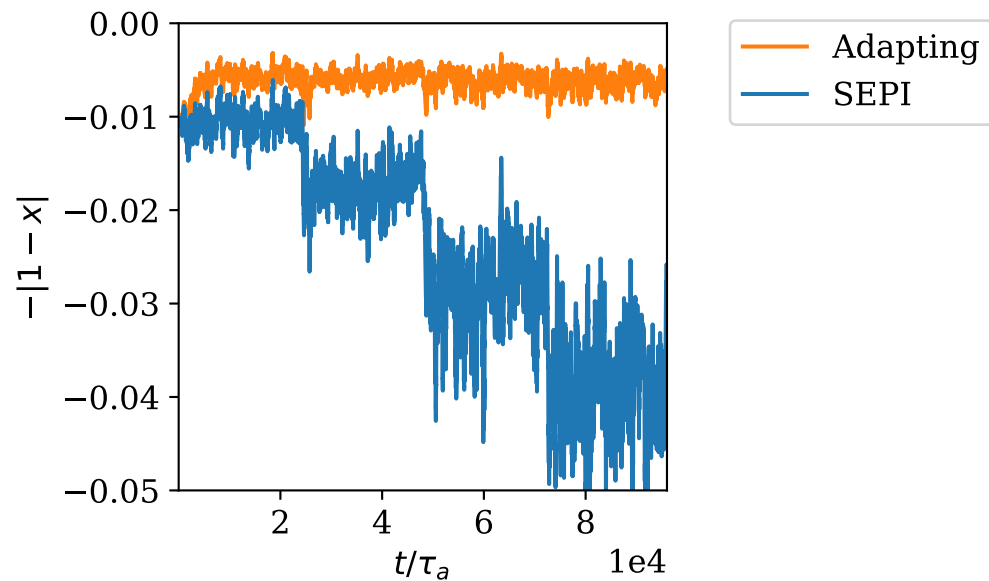

Supplement: Source code 1. [file elife-67455-code1.zip › data_and_scripts/Supplement/S4/FigS4.pdf]

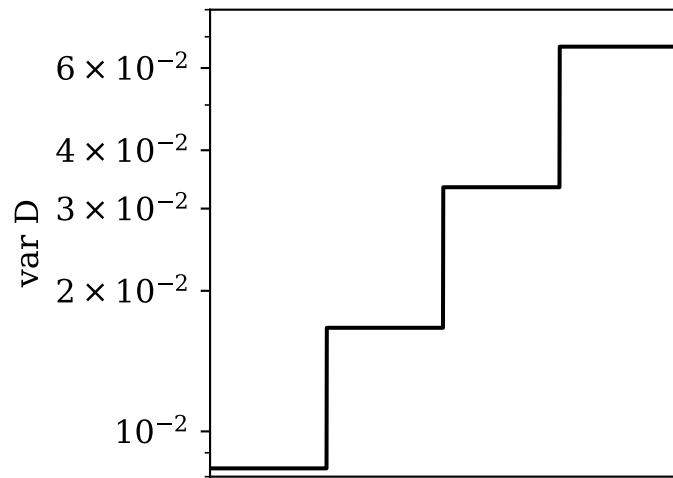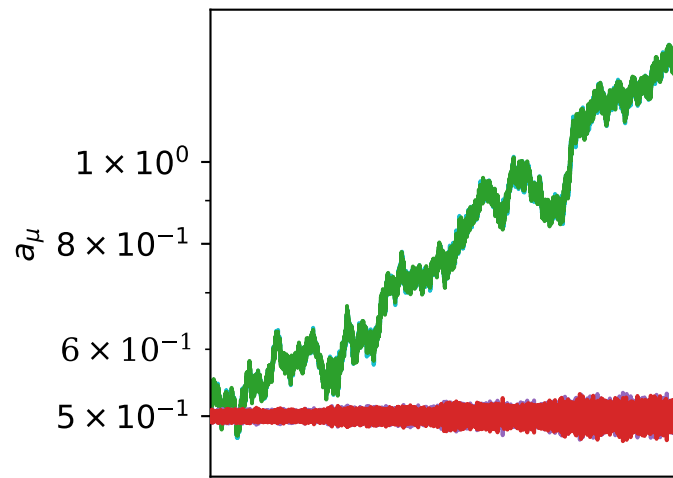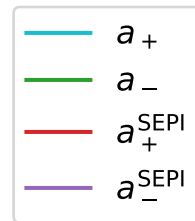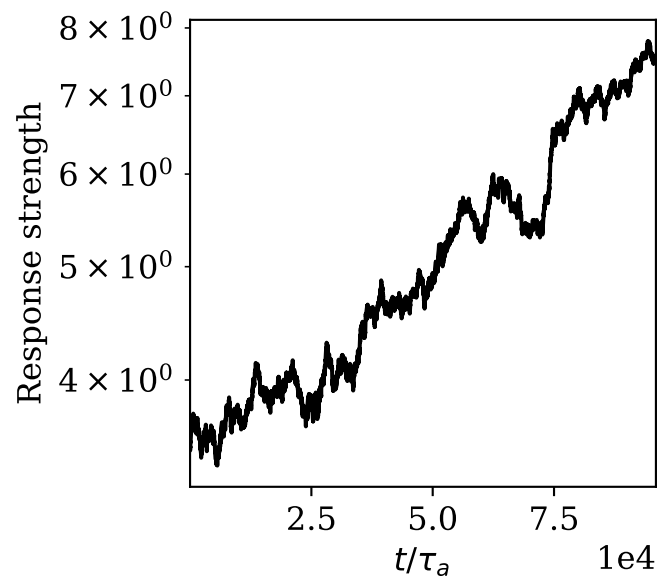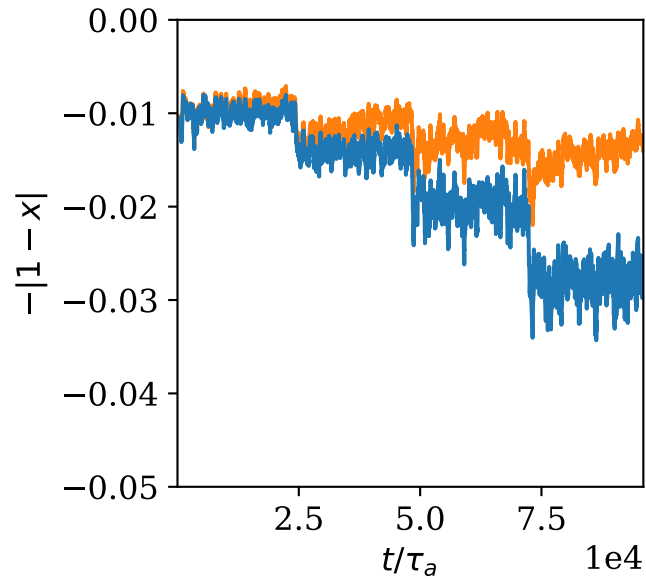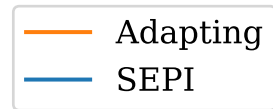

Supplement: Source code 1. [file elife-67455-code1.zip › data_and_scripts/Supplement/S6/figS6.pdf]
